# An Investigative Study on the Structural, Thermal and Mechanical Properties of Clay-Based PVC Polymer Composite Films

**DOI:** 10.3390/polym15081922

**Published:** 2023-04-18

**Authors:** Neeraj Kumari, Chandra Mohan, Arvind Negi

**Affiliations:** 1 Department of Chemistry, SBAS, K. R. Mangalam University, Gurugram 122103, India; 2 Faculty of Pharmacy, DIT University, Dehradun 248009, India

**Keywords:** solution casting, clay, polyvinyl chloride, thermal analysis, tensile strength, hardness

## Abstract

The present study aims to explore the impact of pristine and surfactant-modified clays (montmorillonite, bentonite and vermiculite) on the thermomechanical properties of a poly (vinyl chloride) (PVC) polymer film. Initially, clay was modified by employing the ion exchange method. The modification of clay minerals was confirmed by the XRD pattern and thermogravimetric analysis. Pristine PVC polymer film and clay (montmorillonite, bentonite and vermiculite)-based PVC polymer composite films were fabricated using solution casting. The ideal dispersion of surfactant-modified organo-clays was observed in the PVC polymer matrix due to the hydrophobic nature of modified clays. The resultant pure polymer film and clay polymer composite film were characterized using XRD and TGA, and their mechanical properties were determined using a tensile strength tester and Durometer. From the XRD pattern, the intercalation of the PVC polymer film was found in the interlayer of organo-clay while exfoliation or partial intercalation and exfoliation were observed for pristine clay mineral-based PVC polymer composite films. Thermal analysis indicated a lowering of the decomposition temperature of the composite film as clay promotes the thermal degradation temperature of PVC. Improvement in the tensile strength and hardness was found to be more frequent in the case of organo-clay-based PVC polymer films, which is only due to the hydrophobic nature of organ clays, resulting in greater compatibility with the polymer matrix.

## 1. Introduction

A class of hybrid materials with layered silicate minerals that are inorganic fillers and an organic polymer matrix is known as a polymer nanocomposite. Reinforcing the inorganic filler in the polymer matrix is one of the most prominent methods that can improve the physicochemical properties of the polymer matrix, which is formed by the dispersion of a known amount of clay into a pristine polymer matrix [1]. In recent years, a lot of work has been performed to continue the research of new composites based on these clay minerals based on the findings of Toyota researchers who used layered silicates as reinforcing fillers for various thermoset and thermoplastic polymers. Properties such as thermal behavior, barrier performance, mechanical, heat distortion temperature, flame retardance, gas permeability, solvent resistance and others can be enhanced by dispersing just a little amount of nanoclay into the polymer matrix. In nanocomposites, a large area forms at the interface between nanoclay and polymer matrix, as compared to normal composites, due to which the properties of nanocomposites are enhanced to a greater extent. Due to their unique characteristics and improved properties, polymer nanocomposites are also known as advanced structural materials [2,3,4,5,6,7].

Different types of clay minerals, such as kaolinite, laponite, vermiculite and smectite, have been used as reinforcing agents due to their specific characteristics. Among all the clay minerals, smectite (montmorillonite (Mt) and bentonite (Bent)) and vermiculite (Vt) clay minerals have been used on a large scale due to their easy availability, high cation exchange capacity, high specific surface area, low cost, and environmentfriendly nature. Both types of naturally occurring clay minerals belong to a 2:1 group, where an octahedral sheet is sandwiched between two tetrahedral sheets. They are also known as the aluminosilicate layer. All clay minerals show negatively charged surfaces due to the isomorphic exchange of Al^3+^ for Si^4+^ in the tetrahedral layer and Mg^2+^ for Al^3+^ in the octahedral layer. The negative charge of clay minerals is neutralized by the interlayer ions such as Na^+^, K^+^, Mg^2+^, and Ca^2+^. These ions are mainly responsible for the hydrophilic nature of clay minerals [6].

It is not possible to have a complete dispersion of clay in the polymer matrix as the polymer matrix has a hydrophobic nature, while pristine clays (such as montmorillonite, bentonite, and vermiculite) are hydrophilic in nature [8]. Various approaches have been used to enhance clay and polymer compatibility. Therefore, the clay modification process using organic moieties has been used on a large scale to produce structured polymer nanocomposites. This is one of the best methods to enhance compatibility, which is via the modification of the surface of clay minerals [9].

There are several ways to modify clay minerals, but the ion exchange method using cationic surfactants is one of the most common methods [10], where the intercalation of the cationic surfactant into the interlayer of clay minerals is possible by replacing the interlayer’s inorganic ions, rendering the clay surface organophilic. The modification of clay minerals is performed with phosphonium ions, quaternary ammonium, and imidazolium ions by substituting inorganic exchangeable ions. Incorporating modified clays into a polymer matrix improves the interfacial adhesion, which enhances compatibility [11,12].

During the fabrication of nanocomposites, the confinement of the polymer chain was observed, and the confinement of the nanometer space generally affects the interaction between polymer chains. Three methodologies are used for the fabrication of clay-based polymer nanocomposites: solution casting, melt blending/processing and in situ polymerization [13,14,15,16,17,18].

Two types of layered silicate/polymer structures are obtained: intercalated and exfoliated [19,20,21]. Intercalated structure: The intercalation of a single extended polymer chain was observed between the layers of the silicate minerals, resulting in a well-ordered multiplayer morphology. Exfoliated structure: The complete exfoliation of silicate layers (individual layers) is observed. Generally, an intercalated or exfoliated structure of clay polymer nanocomposites depends on the chemistry of the entropic and enthalpic factors. There are many aspects of applications of clay minerals, for example, increments in the thermal stability of elastomer blends by adding nanoclay, the enhancement of the transport properties of natural rubber by incorporation of nanostructured Na-bentonite, and the improvement in the stress relaxation of natural rubber by incorporation of organo-montmorillonite. The better enhancement of the mechanical properties and thermal degradation temperature of PP-based nanocomposites obtained from quaternary ammonium modified montmorillonite. The thermos-oxidative stability of polypropylene and polyethylene was enhanced after the incorporation of carbon nanotube (CNT) and organo-montmorillonite [22]. Araujo reported a comparative study of organo-montmorillonite-based polyethylene composites where montmorillonite was modified using four different types of ammonium salts and confirmed the enhancement in various characteristics such as thermal stability, flammability resistance and mechanical properties [23].

Plenty of attention has been given by scientists and researchers concerning the preparation of clay-based polyvinyl chloride (PVC) polymer films. PVC is the most common polymer and is used on a large scale due to its unique characteristics, such as ease of processing and remarkable optical and flammable properties compared to other polymers, such as polyamide (PA), polymethyl methacrylate (PMMA), polyethylene (PE), polypropylene (PP) and many other polymers. However, due to some inherent shortcomings of PVC polymers, such as low thermal stability and brittleness, the application of PVC is limited. Therefore, it is required to produce a modified PVC polymer with remarkable properties for wide applications and high added values. Due to their enhanced properties and unique characteristics, clay-based PVC polymer composites are used for wide applications, such as coating, automotive parts [24,25]. The enhancement of the physicochemical properties of PVC polymer films is attained with the loading of low concentrations of clay/modified clay (organo-clay) [26,27,28,29,30].

There are many researchers who have confirmed improvements in the properties of PVC polymer composites after the dispersion of clay/modified clay, but none of them have ever explained the effect of different types of clay minerals on the properties of a PVC polymer in a comparative study. Wan et al. synthesized montmorillonite-based PVC nanocomposites using a melt blending method and confirmed the partially intercalated structure of nanocomposites with enhanced properties [31]. Na-bentonite-based PVC polymer nanocomposites were synthesized via in situ polymerization and they exhibited an improved thermal stability, transparency and shear-thinning rheology [32]. Wang and his co-workers used the melt blending method to synthesize intercalated montmorillonite-based PVC polymer nanocomposites and studied the thermal and mechanical properties of the synthesized nanocomposites [24].

Lots of research articles have been reported about the synthesized clay-based PVC polymer nanocomposites through melt blending in situ polymerization methods [33,34] but there are only a few papers that have investigated the synthesis of PVC polymer nanocomposites through a solution casting method.

The present paper is directed toward the preparation of clay/organo-clay-based PVC polymer films through the solution casting method and the investigation of their thermal and mechanical properties. The effect of different types of clay (montmorillonite (Mt), bentonite (Bent) and vermiculite (Vt)) is also studied on the structural and thermo-mechanical properties of PVC polymer films. Initially, pristine clay was modified using the cation surfactant, cetylpyridinium chloride (CPC), through an ion exchange method. The modified clays are known as organo-clays. The modified clays, also known as organo-clays, are further dispersed into PVC polymers to fabricate PVC polymer films. The main purpose of the present paper is to explore the effect of various clay/modified clay (Mt, Bent and Vt) on the structural, thermal and mechanical properties of PVC polymer film.

## 2. Materials and Methods

All types of clay minerals (Na-montmorillonite, Ca-nano bentonite (<80 nm particle size) and Mg-vermiculite) were procured from Sigma Aldrich Chemical Pvt. Ltd., St. Louis, MO, USA. Cetylpyridinium chloride and tetrahydrofuran (THF) were obtained from E. Merck Pvt. Ltd., Mumbai, India. Poly (vinyl chloride) (PVC) was obtained from Taj Resin Chemical Pvt. Ltd., Delhi, India.

### 2.1. Modification of Clay Minerals Using Cation Surfactant

The modification of clay minerals was carried out through ion exchange method using cetylpyridinium chloride (CPC), a cationic surfactant, by modifying the reported procedure [28]. A known amount of pristine Mt was added to 400 mL of double-distilled water and then stirred for 24 h. A total of 100 mL of cationic surfactant (2%) solution was added into the clay suspension under continuous stirring and further stirred for 2 h. The resultant solution was centrifuged for 20 min at 8000 rpm to separate supernatant and residue. The residues were allowed to dry at room temperature and were then crushed in pestle and mortar to acquire the fine particles.

The modification of bentonite and vermiculite was performed by following the same procedure as mentioned above.

The resultant powders are also called organo-montmorillonite (OMt), organo-bentonite (OBent) and organo-vermiculite (OVt).

### 2.2. Synthesis of PVC Polymer Film

Pristine PVC polymer film was simply synthesized through solution casting method. Initially, 5 g of PVC polymer was dissolved using 25 mL of tetrahydrofuran solvent under constant stirring at room temperature until a homogenous solution was obtained. The main reason for using this solvent is its unique characteristics. It has the capacity to dissolve PVC polymers at 20 °C, while other solvents, such as cyclohexanone and cyclopentanone, do so at 40 °C [35]. The solution was poured into a Petri dish and kept overnight to obtain a solvent-free film.

### 2.3. Synthesis of Clay-Based PVC Polymer Composite Film

For the synthesis of clay-based PVC polymer composite films, initially, PVC polymer was dissolved in tetrahydrofuran solvent under magnetic stirring. As reported, water is an excellent swelling agent for Mt, but it is a very poor solvent for the organo. Peterson et al. confirmed that THF is an excellent solvent for most of the modifiers but is quite a poor swelling agent for Mt [36].

On complete dissolution of polymer, clay/organo-clay (relative to the weight of polymer) was added as inorganic filler into the polymer solution and stirred until complete dispersion of clay was obtained. The solution containing clay/organo-clay and polymer was poured into a Petri dish and left overnight to evaporate the obtained solvent and polymer film. The whole synthesis procedure was performed at room temperature.

Interventional studies involving animals or humans and other studies that require ethical approval must list the authority that provided approval and the corresponding ethical approval code.

## 3. Results

### 3.1. Surface Charge Analysis

The surface charge analysis was performed using a Malvern Zetasizer nanosize, Malvern Panalytical Ltd., Malvern, UK. To determine the surface charge, 0.01% of clay/organo-clay was dispersed into double-distilled water and allowed for ultrasonication for 20 min, followed by filtering the solution using 25 µm-sized filter paper.

The pristine clay minerals have a negatively charged surface, as mentioned in the Introduction section, which is further verified by the zeta potential value. The zeta potential value is negative for all clay minerals, as shown in Table 1, indicating a negatively charged surface. On interaction with CPC, the surface charge of OMt and OBent becomes positive (+29.3 mV and +31.1 mV), suggesting CPC’s presence on their surfaces. In the case of OVt, since the surface charge remains negative (−19.1 mV), it confirmed the presence of CPC on the surface as the zeta potential value was modified from −47.0 mV to −19.1 mV. Since the Vt has a negligible ion exchange capacity due to the high layer charge density, a restriction on the expansion of interlayer spacing occurs, and so the interaction with CPC was only possible at the surface.

### 3.2. X-ray Diffraction Studies

To investigate the intercalation or surface interaction of CPC with clay minerals and exfoliated or intercalated structure of clay-based PVC polymer composite films. XRD patterns were performed and recorded using a Philips X′ Pert-PRO Panalytical (model 3040160) Davis, CA, USA, between 2θ values of 2° and 30°.

#### 3.2.1. XRD Pattern of Clay before and after Modification

**Figure 1:**
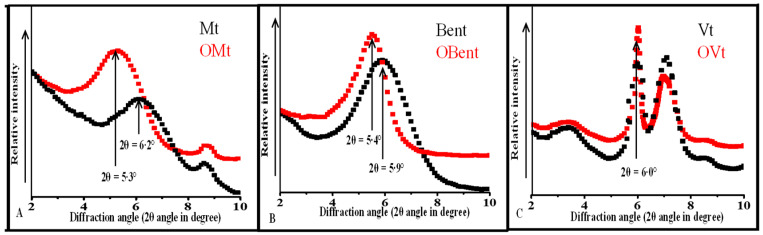
XRD pattern of (**A**) Mt, OMt; (**B**) Bent, OBent; (**C**) Vt, OVt.

The X-ray diffraction method is one of the most prominent methods used to examine the interlayer spacing. The XRD pattern showed the diffraction peak point of Mt 6.2°, with the 2θ value corresponding to the 001 plane and indicating an interlayer spacing, 14.25 Å. After modification with a cationic surfactant, the diffraction peak point was at 2θ value 5.25°, which shifted toward a lower angle. Due to which, the interlayer spacing increased from 14.25 Å to 17.0 Å (Table 2). The expansion in the interlayer spacing of Mt was observed as the CPC intercalated into the interlayer of Mt. An additional peak at 8.7° occurred because of the existence of illite in Mt (Figure 1A).

The diffraction peak point of Bent was at 5.8°, corresponding to the 001 plane and showing 15.24 Å basal spacing. After modification, the diffraction angle moved toward a lower angle, and 5.4° results in an increase in interlayer spacing from 15.24 Å to 16.4 Å (Figure 1B) (Table 2). The increment in the basal spacing confirmed the intercalation of CPC in the interlayer of Bent.

The diffraction peak point for Vt was at 6.0°, indicating 14.72 Å interlayer spacing. There was no significant change in the diffraction peak point of Vt after the interaction. suggesting only a surface interaction (Figure 1C). Therefore, the interlayer spacing of Vt remained the same (Table 2). Two more diffraction peak points were observed at 3.3° (due to the interstratification) and 7° (due to presence of hydrated interlayer ions such as Mg^2+^, K^+^). The negligible change in interlayer spacing of Vt confirmed its limited expansion, suggesting an insignificant ion exchange capacity of Vt.

The expansion of the interlayer spacing was more in the case of Mt compared to Bent, indicating a greater extent of intercalation of CPC in the interlayer of Mt.

**Figure 2:**
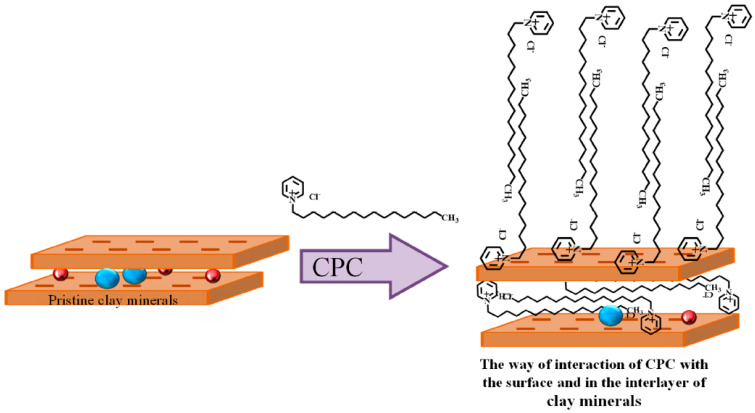
Possible interaction of CPC surfactant with clay minerals.

The possible interaction of CPC with clay monolayers and bilayers is shown in Figure 2.

#### 3.2.2. XRD Pattern of Clay-Based PVC Polymer Composite Film

**Figure 3:**
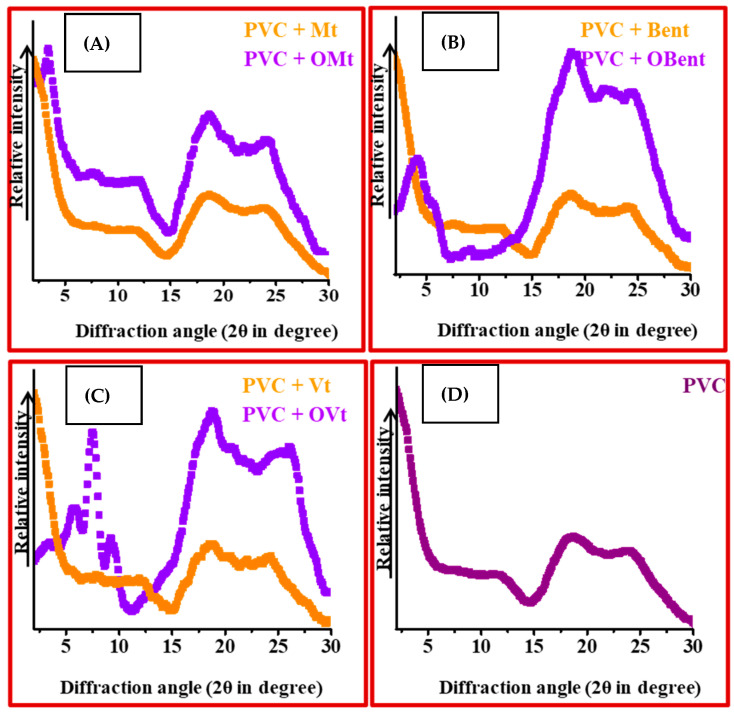
XRD pattern of (**A**) Mt, OMt-based PVC polymer composite film. (**B**) Bent, OBent PVC polymer composite film (**C**) Vt, OVt PVC polymer composite film. (**D**) Pure PVC polymer film.

**Figure 4:**
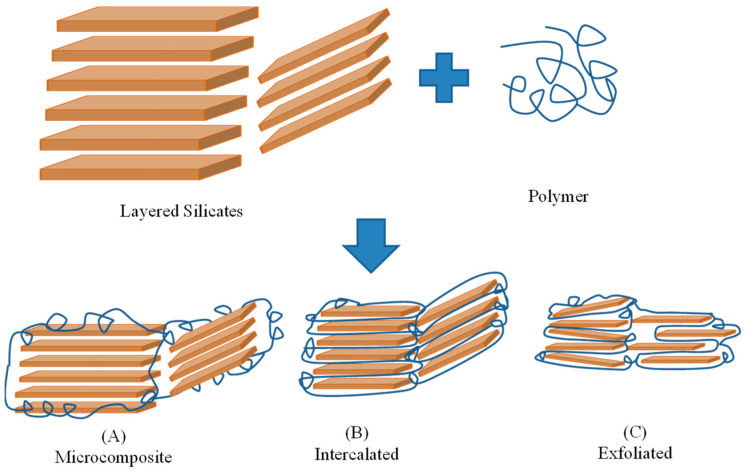
Schematic showing three morphological states for clay-based PVC polymer nanocomposites. (**A**) Microcomposite phase. (**B**) Intercalated phase. (**C**) Exfoliated phase.

Figure 3 shows the XRD patterns of the pristine PVC polymer film, the pristine clay-based polymer composite film and the organo-clay-based PVC polymer composite film. In the case of pristine clay-based PVC polymer composite films, there was no diffraction peak point at Mt, Bent and Vt, suggesting either the exfoliation or partial exfoliation and partial intercalation of the structure of clay-based polymer composite films (Figure 4) [37,38].

XRD pattern of OMt and OBent-based PVC polymer composite film showed shifting of the characteristic diffraction peak point toward lower angle, 3.27° and 4.04° having interlayer spacing of 27.01 Å and 21.87 Å suggesting PVC in the interlayer region of OMt and OBent. The diffraction peak point of OVt practically remained the same for OVt-based PVC polymer composite films, suggesting a microcomposite structure with no dispersion or little dispersion of OVt (Figure 4). There was no significant characteristic diffraction peak of pure PVC polymer films, which was found due to the amorphous nature of the PVC polymer (Figure 3D).

Araujo et al. confirmed the intercalation as well as the exfoliated structure of the clay-based polyamide 6 nanocomposite [29]. Hadj-Hamou and Yahiaoui synthesized antimicrobial poly (ϵ-caprolactone) (PCL), poly (vinyl chloride) (PVC) and organo-clay nanobioblended films through the melt blending method, where the nanocomposite showed a mixed intercalated/partially exfoliated structure [39].

Based on the above observation, it can be concluded that there was a strong interaction between the inorganic filler and PVC polymer.

### 3.3. Thermogravimetric Analysis

The thermal behavior of PVC polymer films and clay-based PVC polymer composite films was analyzed using TGAQ–500, Perkin Elmer in a temperature range of 30–800 °C in a nitrogen atmosphere with a flowof 10 mL/minute at 10 °C/minute rate.

#### 3.3.1. Thermogravimetric Analysis of Clay before and after Modification

**Figure 5:**
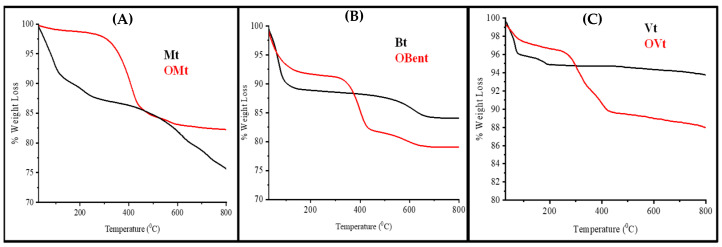
Thermogravimetric analysis (TGA curve) of (**A**) Mt, OMt, (**B**) Bent, OBent and (**C**) Vt, OVt.

**Figure 6:**
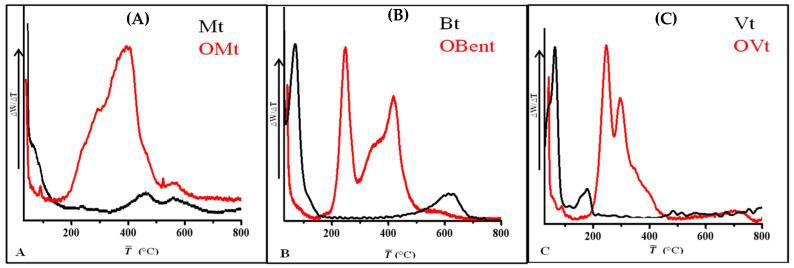
Thermal analysis (DTG curve) of (**A**) Mt, OMt, (**B**) Bent, OBent and (**C**) Vt, OVt.

The thermogram and its derivative for pristine and modified clay is shown in Figure 5 and Figure 6. The first weight loss was observed from 30 °C to 150 °C due to the physically adsorbed water, which is found less in the case of organo-clays, indicating the presence of a cationic surfactant on the surface of clay minerals, which was further confirmed from DTG curves. The second stage of weight loss was from 150 °C to 400 °C. In the case of pristine clay minerals, the weight loss was due to the loss of interlayer water (236 °C for Mt and 178 °C for Vt, confirmed from the DTG curve), while the weight loss in the case of organo-clays indicated the decomposition of CPC in their interlayer or present on the surface As the DTG data showed weight loss at 400 °C for OMt and 395 °C for OBent due to the decomposition of CPC present in the interlayer.

At the third stage, the weight loss from 400 to 800 °C was due to dehydroxylation of the structural OH groups (465 °C and 560 °C for Mt and 620 °C for Bent). In the case of Bent, no peak was observed between 150 °C and 400 °C, indicating no thermally induced changes.

#### 3.3.2. Thermogravimetric Analysis of Clay-Based PVC Polymer Film

**Figure 7:**
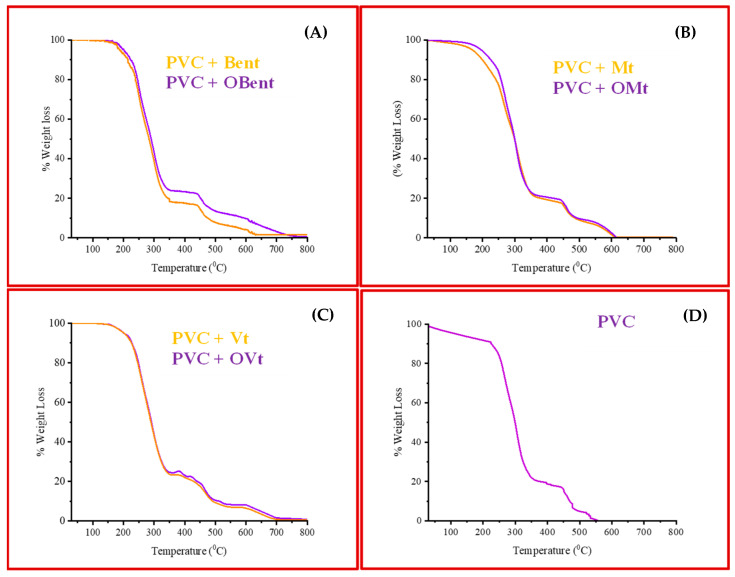
Thermogravimetric analysis (TGA curve) of (**A**) Mt, OMt-based PVC polymer composite, (**B**) Bent, OBent PVC polymer composite film, (**C**) Vt, OVt PVC polymer composite film and (**D**) pure PVC polymer film.

**Figure 8:**
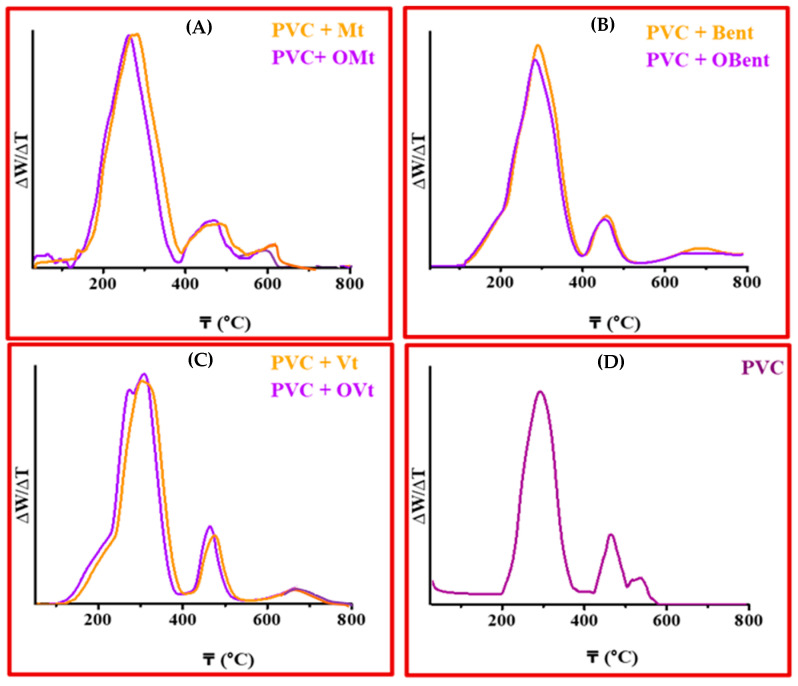
Thermogravimetric analysis (DTG curve) of (**A**) Mt, OMt-based PVC polymer composite, (**B**) Bent, OBent PVC polymer composite film, (**C**) Vt, OVt PVC polymer composite film and (**D**) pure PVC polymer film.

Figure 7 and Figure 8 show the thermal behavior of PVC polymer films containing pristine clays and organo-clays. The thermal degradation of the pure PVC took place in two major steps. The first decomposition observed in the range of 220–350 °C (294 °C confirmed from the DTG curve) was due to the dehydrochlorination of the polymer chain and is known as onset decomposition (Tonset). The second decomposition occurred at a high temperature range from 450 °C onward (465 °C and 535 °C), which was due to the cyclization of conjugated polyenes sequence in PVC to form the aromatic compounds with the residue char; this stage is known as the fastest decomposition (Trpd) [40].

The decomposition temperature of PVC was found to be high in both stages after the dispersal of pristine/organo-Mt, -Bent and -Vt in the PVC polymer matrix. Clay in the PVC matrix results in the development of char and inhibits the distribution of volatile products, which results in an increase in the thermal stability.

In the case of OMt-, OBent- and OVt-based PVC polymer composite films, the onset temperature decreased in the range of 220–350 °C (281 °C in OMt—PVC; 275 °C in OBent—PVC; 272 °C in OVt—PVC polymer composite film) due to the influence of ammonium salt, which has a strong interaction with the chloride of PVC. The organo-clay acts as a catalyst and enhances the dechlorination of PVC. Trpd for organo-clay-based PVC polymer composite films was high since they act as a barrier to prevent the evaporation of small molecules, which are produced during the thermal decomposition of PVC. In the case of clay-based PVC polymer composite films, the presence of clay enhanced the char formation, which resulted in a hindrance to volatile product diffusion. Furthermore, the amount of residue in the case of clay-based composite films is high, indicating the presence of clay in the PVC matrix [41,42].

The mass loss was found to be lower (3–5%) between 250 °C and 300 °C for pristine clay/organo-clay-based PVC polymer composite films. Mondragon et al. also confirmed the decrease in the thermal stability of PVC polymers after treating them with organo-montmorillonite at 10 °C, suggesting that organo-montmorillonite promotes the thermal degradation of PVC [43,44,45]. Chee and Jawaid also showed the effect of montmorillonite on the morphology, thermal and mechanical properties of epoxy/organo-clay nanocomposites, where they confirmed the lower decomposition temperature of nanocomposites compared to the pristine epoxy. The lowering of thermal stability was due to the existence of organic moieties on the surface of the clay, which resulted in a lowering of thermal stability [46,47].

### 3.4. Morphological Study

Surface images of synthesized samples were recorded using scanning electron microscopic technique. One drop of the aqueous dispersion of samples was mounted on stubs, air-dried and sputter-coated with gold in a vacuum evaporator and photographed using a scanning electron microscope model (ZEISS EVO 40) with an accelerating voltage of 30 KV coupled.

#### 3.4.1. Morphological Studies of Clay before and after Modification

**Figure 9:**
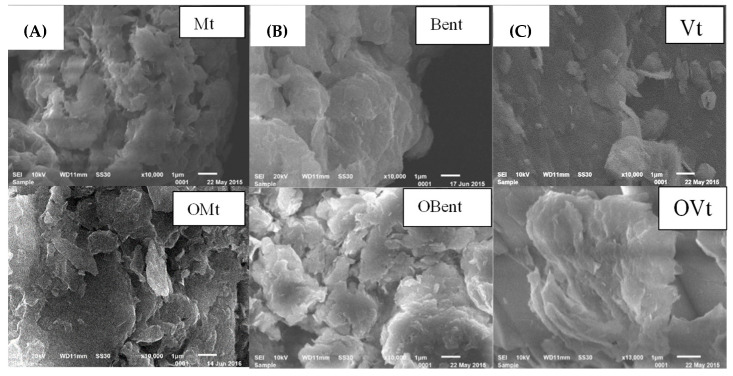
SEM images of (**A**) Mt, OMt, (**B**) Bent, OBent and (**C**) Vt, OVt.

An SEM is mainly performed to analyze the changes in the morphology of pristine and modified clay minerals. The pristine clay minerals have massive plates with some phase separation. Due to the intermolecular forces, the particles in pristine clay minerals are closely attached and present in their aggregated form. On interaction with the cationic surfactant, there is a significant change in the morphology. Due to the increase in the interlayer spacing of clay, more voids can be seen to have small and aggregated particles (Figure 9). The layers of vermiculite are weakly compacted into polygonal sheets with flaked borders, whereas in the case of OVt, a distinctive, porous microstructure was observed.

#### 3.4.2. Morphological Studies PVC Polymer Films and their Composite Films

**Figure 10:**
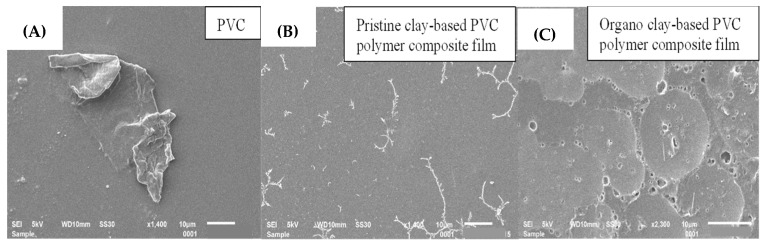
SEM images of (**A**) pure PVC polymer film, (**B**) clay-based PVC polymer composite film and (**C**) organo-clay-based PVC polymer composite film.

Figure 10 shows the morphology of pure PVC polymer films and pristine clay/organo-clay-based PVC polymer composite films. From the SEM image, it is clear that the pure PVC film has a fractured surface. A significant change is observed in the morphology of the polymer film after the dispersion of the inorganic filler. The bright white spot present on the surface of the composite film confirms the presence of clay/organo-clay, whether it is in dispersed or aggregated form. In the case of organo-clay-based polymer films, the fractured surface is clearer, indicating a brittle material, as the presence of organo-clay forces the crack to follow a more complex path, increasing the fracture surface area of the film.

### 3.5. Mechanical Properties

#### 3.5.1. Tensile Strength and Young’s Modulus of Clay-Based Polymer Composite Films

The tensile strength and Young’s modulus of pristine PVC polymer films and clay-based PVC polymer composite films were determined using a tensile testing machine (Instron UTM 3369). The polymer film sample was exposed to a tensile load with a 10 mm/min cross speed after being held between the upper and lower jaw of the tensile tester. The tensile strength was calculated using the following formula:

(1) $$Tensile\;strength=\frac{Force(maxiumu\;load\;applied\;to\;elongate\;the\;film)}{Area(width\times thickness)}$$

Table 3 showed the tensile strength and Young’s modulus properties of pristine PVC polymer films and clay/organo-clay-based PVC polymer composite films. The enhancement in the tensile strength of the PVC was observed by a factor of 1.4, 1.6 and 1.3 on the dispersal of pristine Mt, Bent and Vt, respectively. The dispersion of organo-Mt, -Bent and -Vt further improved the tensile strength of the organo-clay-based PVC polymer composite film by 1.7, 1.8 and 1.3 factor, respectively.

The Young’s modulus of PVC improved after reinforcing the inorganic fillers, which indicated their improved firmness. The Young’s modulus was increased by a factor of 1.1 (Mt), 1.2 (Bent) and 1.0 (Vt) after incorporation of pristine clay, while in the case of organo-clay, it was improved by a factor 1.2 (OMt), 1.4 (OBent) and 1.0 (OVt), respectively.

The tensile strength and Young’s modulus of Vt and OVt-based PVC polymer composite films remain the same, as there was no intercalation of CPC in the interlayer of Vt, which was confirmed by the XRD and thermal analysis.

The improvement in the tensile strength suggested that organo-clay-based PVC polymer composite films show better resistance against the tensile stress due to their better compatibility with hydrophobic PVC polymers, which is acquired before the deformation of their shape.

#### 3.5.2. Hardness

The hardness of PVC polymer films was enhanced by a factor of 1.127, 1.181 and 1.127 after reinforcing pristine clay Mt, Bent and Vt, respectively as shown in Table 4. The dispersion of organo-clay, OMt, OBent and OVt improved the hardness by factors of 1.363, 1.272 and 1.145, respectively. Due to the hydrophobic nature and better compatibility of organo-clays, they were well dispersed in the polymer matrix and enhanced the mechanical properties of the polymer matrix by acting as a reinforcement material [48]. The improvement in hardness is an indication of better resistance against the deformation of the polymer film on the application of a compressive force. Kumar et al. also confirmed the enhancement in the hardness of clay-based PMMA polymer nanocomposites. The hardness of pristine PMMA polymer composites was 58 and enhanced by a factor of 1.34 (78) after treating the PMMA polymer with organo-clay and a compatibilizer, as clay platelets enhanced the hardness by restricting the indentation [49,50].

Based on the above observation, it can be said that the interactions of PVC with organo-Mt, -Bent and -Vt show a better improvement in the mechanical properties (tensile strength, Young’s modulus and hardness) of PVC polymer films. The better improvement in the mechanical properties of organo-clay-based PVC polymer composite films is due to the inherently high moduli and aspect ratio of organo-clay results in inducing more surface area with a polymer matrix. Furthermore, clay acts as a stress transfer agent after dispersing in the polymer matrix results in enhancement in the mechanical properties.

## 4. Conclusions and Future Prospects

In this study, the surface of pristine clay minerals was successfully modified using a cationic surfactant, CPC, through ion exchange. The interaction between the cationic surfactant and the clay minerals was confirmed by XRD and thermal analysis. The zeta potential value confirmed the positively charged surfaces of organo-Mt and -Bent due to the presence of CPC in the form of a double layer on their surfaces, while the surface charge of Vt remained negative, indicating a single layer of CPC on its surface. The thermal analysis confirmed the improved thermal stability of clay minerals after modification.

A comparative study was performed to investigate the impact of pristine clay and organo-clays on the structural, thermal and mechanical properties of PVC polymer films. XRD studies confirmed the exfoliation structure of pristine clay-based PVC polymer composite films; intercalation was observed for organo-Mt/Bent-based PVC polymer composite films, while in the case of Vt-based PVC polymer composite films, the microstructural composite was formed. Organo-clay-based PVC polymer composite films showed more enhanced mechanical properties (tensile strength, Young’s modulus and hardness) than pristine clay-based PVC polymer composite films, as organo-clays are hydrophobic in nature; therefore, they demonstrate greater compatibility with hydrophobic PVC polymer, which results in a more homogeneous dispersal into the polymeric matrix. It can also be said that after the modification of the clay surface, the interfacial adhesion between the clay and polymer increased the results in terms of the enhancement of the mechanical properties of PVC polymer composite films.

It is noteworthy that the thermal stability of clay-based PVC polymer composite films needs to be improved as the onset temperature decreases due to the influence of ammonium salt, which has a strong interaction with chloride in PVC. Therefore, investigations must be carried out to identify an alternate to a surfactant that will provide a better stabilizing effect for PVC. An investigative study should be carried out to identify the presence of solvents in polymer composite films, as THF has the ability to intercalate into the interlayer of clay minerals.

## Figures and Tables

**Table 1:** Surface charge of pristine and organo-clays.

Clay	Sample	Zeta Potential mV
Montmorillinite	Mt	−17.60
	OMt	+29.30
Bentonite	Bent	−31.10
	OBent	+35.70
Vermiculite	Vt	−47.00
	OVt	−19.10

**Table 2:** XRD diffraction peak and basal spacing of clay and modified clay.

Clay	Sample	2θ Value	Basal Spacing (d) Å
Montmorillinite	Mt	6.2	14.45
	OMt	5.25	17.00
Bentonite	Bent	5.8	15.24
	OBent	5.4	16.4
Vermiculite	Vt	6.0	14.72
	OVt	5.95	15.09

**Table 3:** Tensile strength and Young’s modulus of pristine and organo-clay-based PVC polymer composite film.

S. No.	Sample Code	Stress (Pascal)	Strain (mm/mm)	Tensile Strength (MPa)	Young’s Modulus (MPa)
01	PVC	07.85	3.55	1.58	0.881
02	PVC + Mt	14.87	4.96	2.20	0.996
03	PVC + OMt	14.28	6.11	2.71	1.072
04	PVC + Bent	16.27	5.70	2.53	0.989
05	PVC + OBent	20.65	6.56	2.91	1.191
06	PVC + Vt	08.87	4.77	2.12	0.898
07	PVC + OVt	14.87	4.93	2.19	0.929

**Table 4:** Hardness of PVC polymer films and clay PVC polymer composite films.

S. No.	Sample Code	Hardness D—Shore
1	PVC	55
2	PVC + Mt	62
3	PVC + OMt	72
4	PVC + Bent	65
5	PVC + OBent	70
6	PVC + Vt	62
7	PVC + OVt	63

## Data Availability

Not applicable.
